# Quantified evaluation of tracheal compression in pediatric complex congenital vascular ring by computed tomography

**DOI:** 10.1038/s41598-018-29071-9

**Published:** 2018-07-25

**Authors:** Rong Xu, Ke Shi, Zhi-gang Yang, Kai-yue Diao, Qin zhao, Hua-yan Xu, Ying-kun Guo

**Affiliations:** 1 Department of Radiology, West China Hospital, Sichuan University, 37# Guo Xue Xiang, Chengdu, Sichuan 610041 China; 2 Department of Radiology, West China Second University Hospital, Sichuan University, 20# Section 3 south Renmin Road, Chengdu, Sichuan 610041 China

## Abstract

Clinically, early diagnosis and treatment is important for survival of pediatric with vascular ring (VR) associated with congenital heart disease (CHD), and accurate evaluation of VR is a prerequisite for repair surgical. The study aimed to assess the quantitative characteristics of tracheal compression in pediatrics with VR and CHD using dual-source computed tomography (DSCT), and further provided effective information for surgical decisions. A total of 49 VR patients with CHD and 56 controls were enrolled. The tracheal quantitative measurements (short diameter, long diameter, tracheal area and tracheal length) were obtained, and the degree of tracheal compression was assessed. Our results indicated that VR associated with CHD may cause more serious and complex symptoms, and the greater tracheal compression were found on DSCT when more severe symptoms were present (*r* = 0.84). The degree of tracheal compression was significantly different within the VR group between those with and without surgery (*P* = 0.002). Finally, there were good agreement among (1-long diameter ratio), (1-short diameter ratio) and (1-area ratio) in patients and controls, respectively. This study indicated that DSCT enables provides accurate quantitative tracheal compression information for VR pediatrics associated with CHD, and evaluation of the degree of tracheal compression by 1-area ratio may contribute to the repair surgical of VR.

## Introduction

Vascular ring (VR) anomalies are rare malformations of thoracic arch-derived vascular and ligamentous structures, and represent approximately 1% of congenital cardiovascular anomalies. The term was first used by Gross to describe a ring of blood vessels encircling the esophagus and trachea^1^. Clinically, isolated VR occasionally may lead to respiratory or gastrointestinal symptoms and some types do not require surgical intervention^2^. However, VR associated with other congenital heart disease (CHD) that may cause serious long-term clinical symptoms, such as respiratory and circulatory dysfunction, the outcome, mortality and complications by year may be due to different form operations and therapy time^2–4^. Thus, early diagnosis and treatment is important for the survival of VR pediatric patients with CHD, and accurate evaluation of VR is a prerequisite for the surgical repair.

Conventionally, several technologies were utilized to assess VR, including echocardiography, computed tomography (CT), and magnetic resonance imaging (MRI)^5–8^. Previous studies showed that multi-detector CT and MRI were able to accurately evaluate vascular anomalies^6^. However, MRI requires a longer scan time, partly limiting its application in vessels of pediatrics^2^. Recently, dual-source computed tomography (DSCT) has played an increasingly important role in the diagnosis of complex congenital heart disease (CHD) due to its fast scan speed, and high image quality^9–11^. To the best of our knowledge, studies that focus on quantitatively evaluating VR-associated tracheal stenosis in pediatrics with CHD using DSCT and the relationship between compression and surgery are lacking^12–14^. Therefore, we investigated the quantitative characteristics of tracheal stenosis in VR pediatric patients with CHD using DSCT, and further provided effective imaging information for surgical decisions.

## Result

### Baseline characteristics

The age, gender, height, weight, and BSA of the VR patients and controls were not significantly different (all P > 0.05). All patients complained of clinical symptoms (Table 1), including recurrent infections (85.7%), cyanosis (69.4%), dyspnea (30.6%), wheezing (16.3%), dysphagia (14.3%), and stridor (10.2%).

**Table 1:** Baseline characteristics of VR patients and controls.

	Vascular ring (n = 49)	Controls (n = 56)	P value
Age (month)	25.31 ± 33.15	31.01 ± 34.50	0.26
Gender (male, %)	46.94	53.57	0.19
Height (cm)	78.74 ± 17.26	81.17 ± 17.44	0.58
Weight (kg)	10.75 ± 5.27	10.56 ± 6.14	0.63
BSA (m²)	0.46 ± 0.17	0.48 ± 0.18	0.61
Heart rate (bpm)	112.01 ± 20.82	121.2 ± 16.51	0.32
Systolic blood pressure (mmHg)	101.37 ± 11.12	95.97 ± 15.68	0.29
Diastolic blood pressure (mmHg)	60.74 ± 11.12	59.27 ± 10.11	0.53
**Symptoms, n (%)**			
Cyanosis	34(69.4%)	—	—
Dyspnea	15(30.6%)	—	—
Dysphagia	7(14.3%)	—	—
Recurrent infections	42(85.7%)	—	—
Wheezing	8(16.3%)	—	—
Stridor	5(10.2%)	—	—
**Intra-cardiac anomalies**			
The tetralogy of fallot	18	—	—
Patent ductus arteriosus	17	—	—
Atrial septal defect	13	—	—
Ventricular septal defect	11	—	—
Other anomalies	<5	—	—
**Extra-cardiac anomalies**			
Collateral circulation	14	—	—
PLSVC	8	—	—
Coronary artery abnormalities	6	—	—
Other anomalies	<5	—	—

Notes: Values are presented as mean ± SD, P value of <0.05 was considered statistically significant in all analyses. PLSVC: pulmonary left superior vena cava.

All patients had associated intra- or extra-cardiac anomalies as follows (Table 1): Tetralogy of Fallot (TOF, n = 18), patent ductus arteriosus (PDA, n = 17), atrial septal defect (n = 13), ventricular septal defect (VSD, n = 11), and other abnormalities (n < 5). Extra-cardiac malformations presented as collateral circulation (n = 14), left pulmonary superior vena cava (n = 8), coronary artery abnormalities (n = 6), or other pulmonary anomalies (n < 5).

### Type of vascular ring

**Figure 1:**
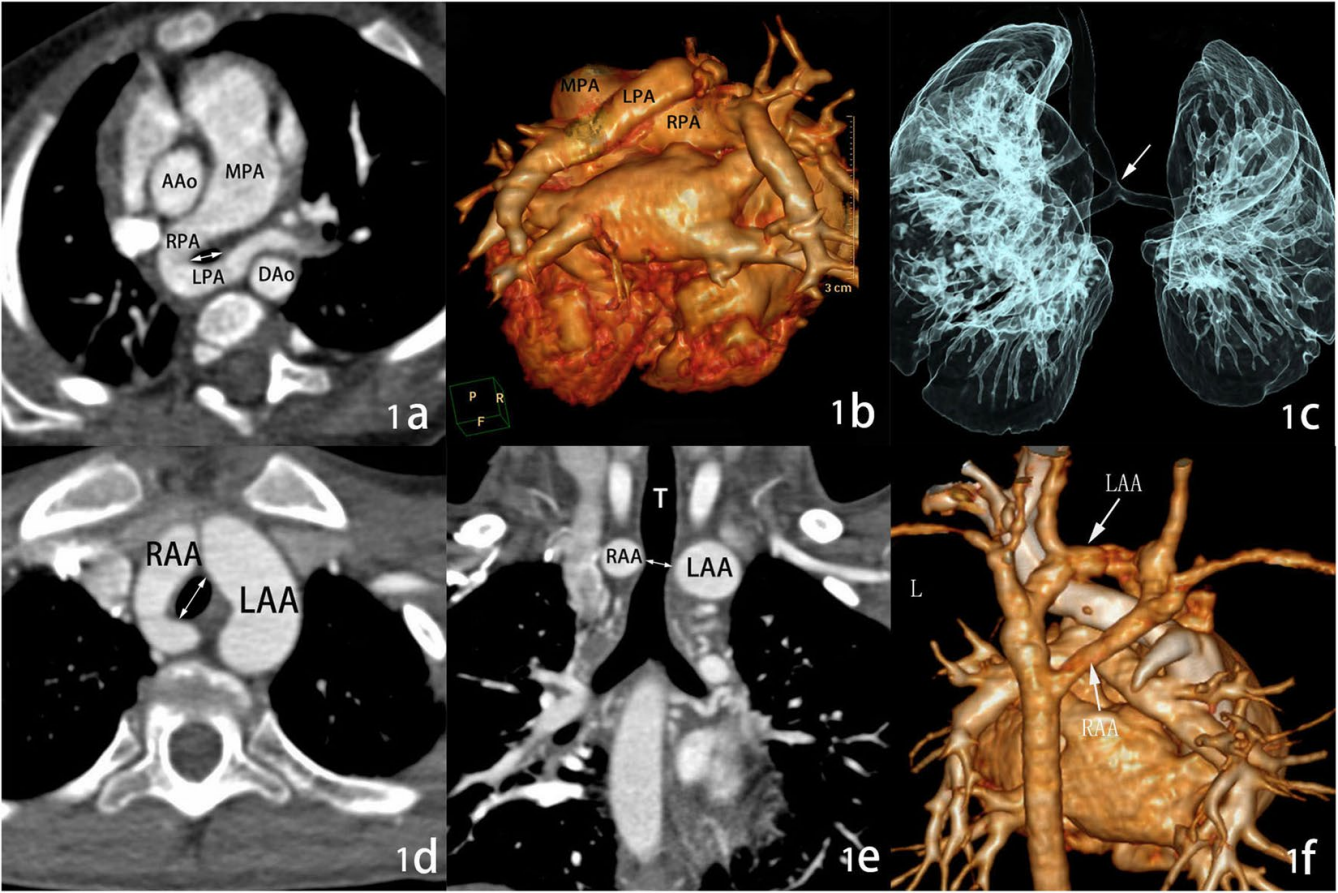
PA sling and DAA on DSCT with MPR. (**a**,**b**) The left pulmonary artery derive from the right pulmonary artery and crossing posterior to the trachea and encircles the distal trachea as it travels to the left lung to form a vascular ring, **(c)** the tracheal stenosis and bridging bronchus (write barrow) were clearly demonstrated; **(d–f)** the left and right aortic arch compress the trachea, causing a regional stenosis (arrow). Abbreviations: MPR, multiplanar reformation, MPA: main pulmonary artery; RPA: right pulmonary artery; LPA: left pulmonary artery; AAo, ascending aorta; DAo, descending aorta; RAA, right aortic arch; LAA, left aortic arch; T, trachea; L, left side.

Of all the 49 patients, five patients (9.7%) had double aortic arch (DAA) completely encircling the trachea and esophagus, and three of these five DAA patients underwent surgical procedures (Fig. 1). Right aortic arch (RAA) with aberrant left subclavian artery (ALSA, n = 11, 21.5%), or with left ductus arteriosus/ligamentum arteriosum (n = 7, 13.7%) presented as incomplete VR. Five RAA patients combined with ALSA and left ductus arteriosus/ligamentum arteriosum that were detected as partial VR. Of 10(19.2%) PA slings, six patients underwent repair surgical. Other uncommon types accounted for less than 10% of cases, and surgical interventions were performed in three of these patients (Table 2).

**Table 2:** Types of vascular ring.

Type	Vascular ring (n = 49)		Total (n)	Percentage (%)	Surgery (n)
	Complete	Partial			
**Double aortic arch**					
Right dominant	4	0	4	7.8	3
Left dominant	1	0	1	1.9	0
**Right aortic arch**					
Aberrant left subclavian artery	0	11	11	21.5	0
LDA/LLA	0	7	7	13.7	0
ALSA+LDA/LLA	1	4	5	9.8	0
Mirror image branching+LDA/LLA	1	4	5	9.8	0
Circumflex artery	0	1	1	1.9	0
Circumflex artery+ALSA	0	3	3	5.8	0
Circumflex artery+ALSA+LDA/LLA	1	0	1	1.9	1
Kommerell’s diverticulum+ALSA	0	2	2	3.9	2
Cervical aortic arch with ALSA	0	1	1	1.9	1
**Pulmonary artery sling**	0	10	10	19.2	6
**Total**	8	43	51	—	13

Notes: ALSA, aberrant left subclavian artery; LDA, left ductus arteriosus; LLA, left ligamentum arteriosum. There are two pulmonary artery sling patients associated with right aortic arch and aberrant left subclavian artery.

### Tracheal assessment

At the level of the tracheal stenosis, tracheal parameters in all VR patients were significantly lower than those of controls (mean bias ± standard deviations): short diameter (0.57 ± 0.19 mm vs. 0.84 ± 0.17 mm, *P* < 0.001), long diameter (0.79 ± 0.29 mm vs. 0.96 ± 0.20 mm, *P* = 0.001), short/long diameter ratio (74.3 ± 14.2% vs. 88.4 ± 7.5%, *P* < 0.001), tracheal area (0.44 ± 0.27 mm² vs. 0.67 ± 0.27 mm², *P* < 0.001). Above the aortic arch level, there were no statistically significant differences in tracheal parameters between VR and control groups (Table 3). The degree of tracheal compression was significantly different within the VR group between the patients with surgery and the patients without surgery. (*P* = 0.002; Table 4).

**Table 3:** The analysis of quantitative tracheal measurement on DSCT.

	Vascular ring (n = 49)	controls (n = 56)	P value
**Tracheal above the aortic**			
Short diameter (mm)	0.78 ± 0.17	0.83 ± 0.18	0.15
Long diameter (mm)	0.95 ± 0.22	0.98 ± 0.19	0.25
Short/long diameter ratio (%)	84.1 ± 7.2	85.3 ± 7.1	0.61
Tracheal area (mm²)	0.64 ± 0.30	0.68 ± 0.27	0.42
**Tracheal stenosis**			
Short diameter (mm)	0.57 ± 0.19	0.84 ± 0.17	<0.001
Long diameter (mm)	0.79 ± 0.29	0.96 ± 0.20	0.001
Short/long diameter ratio (%)	74.3 ± 14.2	88.4 ± 7.5	<0.001
Tracheal area (mm²)	0.44 ± 0.27	0.67 ± 0.27	<0.001
**Tracheal area ratio (%)**	68.2 ± 17.4	98.1 ± 6.0	<0.001
**Total tracheal length (mm)**	4.84 ± 1.25	4.93 ± 1.58	0.74

Notes: Values are presented as mean ± SD. P value of <0.05 was considered statistically significant in all analyses.

**Table 4:** The degree of tracheal stenosis evaluate in patients by DSCT.

Tracheal compression	DSCT		P value
	surgery	no-surgery	
Mild	1	17	P = 0.002
Moderate	7	16	
Severe	5	3	

Notes: P value of <0.05 was considered statistically significant in all analyses.

**Figure 2:**
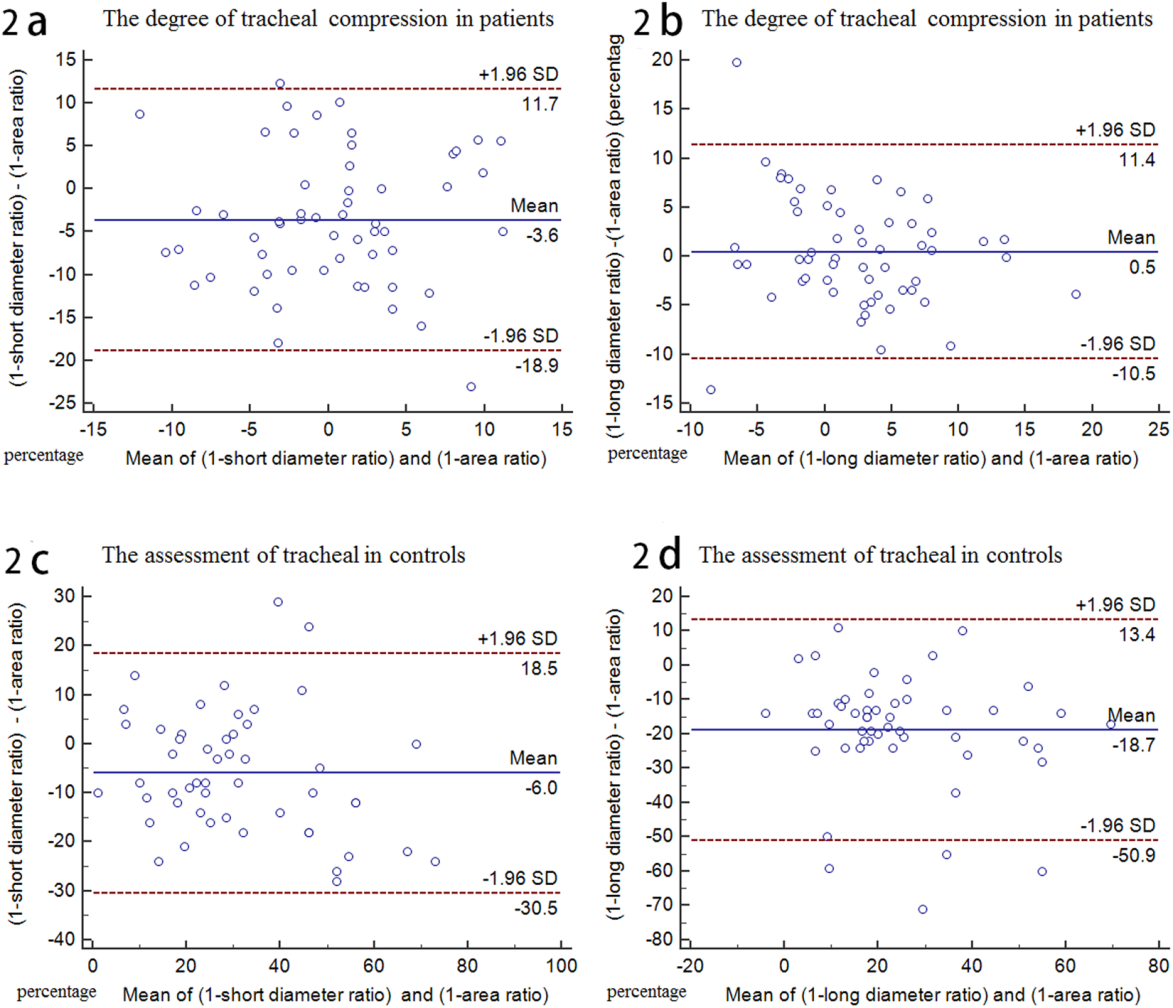
Bland-Altman plots agreement for assessment of the degree of tracheal compression. (**a,b**) The biases between (1-short diameter ratio) and (1-area ratio), (1-long diameter ratio) and (1-area ratio) are 3.6% and 0.5% in patients; (**c,d**) the biases between (1-short diameter ratio) and (1-area ratio), (1-long diameter ratio) and (1-area ratio) are −6.0% and −18.7% in controls.

Bland-Altman analysis demonstrated good agreement between (1-long diameter ratio) and (1-area ratio), (1-short diameter ratio) and (1-area ratio) in patients and controls, respectively (Fig. 2).

### The correlation analysis

**Figure 3:**
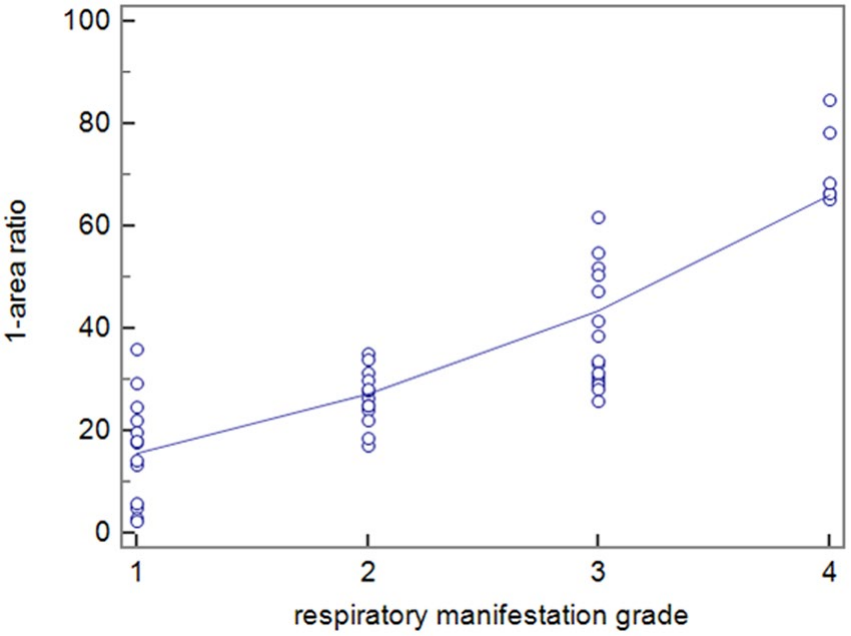
The correlation analysis between tracheal compression and respiratory manifestation grade. The tracheal compression (1-area ratio) was greater in the severe respiratory symptom group (Pearson’s correlation, *r* = 0.84, *p* < 0.001).

There was excellent correlation in the quantitative analysis of tracheal compression (Pearson’s correlation, *r* = 0.84, *P* < 0.001) between (1-area ratio) and respiratory manifestation grade in patients (Fig. 3).

### Reproducibility assessment

The intra- and inter-observer variability of the DSCT measurements was analyzed. The ICCs for intra-observer variability were 0.96 to 0.99, and the ICCs for inter-observer variability were 0.89 to 0.99.

### Radiation dose

DSCT examination was performed with doses as low as reasonably achievable, because the pediatric population is susceptible to ionizing radiation. After we took several measures to reduce the radiation dose, the mean ED of patients was 1.57 ± 1.21 mSv and that of the controls was 1.39 ± 1.37 mSv.

## Discussion

VR encircling the esophagus and trachea by forming a complete or partial constriction that frequently present with heterogeneous clinical signs, such as wheezing, stridor, seal-bark cough, dyspnea, dysphagia, or recurrent infections^3^. Especially in pediatrics, the trachea is particularly vulnerable to extrinsic and results in long-term respiratory problems. As shown in our study, recurrent infections (85.7%), cyanosis (69.4%), and dyspnea (30.6%) were the most common symptoms, and several symptoms often occurred simultaneously, which differed from those of isolated VR^15,16^, indicated that the VR associated with CHD may cause more serious and complex symptoms. In addition, greater tracheal compression was found with DSCT imaging when more severe symptoms were present (*r* = 0.84), the result suggested that in the presence of severe respiratory symptoms, accurate tracheal compression evaluation is essential. Clinically, some types of VR do not need surgical interventions. Nevertheless, surgical repair to effectively relieve compression and improve hemodynamics is inconclusive in VR pediatrics associated with CHD^17,18^. Hyo *et al*.^15^ thought that extrinsic tracheal compression is the most common cause of obstruction during the perioperative period of CHD. Therefore, the evaluation of tracheal compression is extremely important in preoperative.

For clinical practice, echocardiography, CT, and MRI angiography are the major imaging techniques used to identify aortic arch anomalies, and choosing an appropriate imaging modality to assess VR is vital for surgery^8^. Compared with ultrasound and MRI, CT examine can display the airway more clearly for higher density resolution, and the use of DSCT imaging has gained attention recently because of the shorter time, excellent image quality, and more detailed information on cardiovascular structures in the pediatric setting^9^. Moreover, the three-dimensional volumetric acquisitions are particularly useful in the detection of tracheal compression, and definition of vascular relationships, and to display the airway without general anesthesia^6,11^. Hence, we performed this study evaluating VR associated with other cardiovascular diseases by DSCT scan in pediatric patients.

Backer and Mavroudis^2^ defined tracheal stenosis as a reduction in the anatomic luminal diameter of the trachea by more than 50% of the remaining normal trachea. The VR encircles and compresses the trachea and esophagus completely or incompletely, leading to a different degree of tracheal compression. We measured some tracheal-related parameters to evaluate the tracheal compression, and we classified the compression into mild (≤25%), moderate (>25%, ≤50%), and severe (>50%). We found that tracheal compression coexisted in all VR patients with other CHD. Thus, we proposed the hypothesis that the degree of tracheal stenosis may be a determining factor for VR repair treatment. Our data demonstrated that tracheal measurements, such as the short diameter, long diameter, and tracheal area on the tracheal stenosis slices were significantly lower than those of controls. Similar to previous studies^13,17^, our study indicated that tracheal compression usually was caused by VR with CHD, and on the tracheal stenosis slice in imaging, short/long diameter ratio lower than normal controls manifested tracheal anatomic luminal may occur deformation and present as oval or irregular shape. On the contrary, these tracheal parameters above the aortic arch level, in addition to total tracheal length, were not significantly different between subjects with and without VR, indicating that the tracheal development in pediatric with congenital VR may be normal.

Furthermore, for quantitative assessment of the degree of tracheal compression, we demonstrated good agreement between (1-long diameter ratio) and (1-area ratio) and (1-short diameter ratio) and (1-area ratio). And in VR patients underwent vascular surgical intervention, we found the degree of tracheal stenosis were significantly greater than that in non-surgical patients. These findings indicated that the tracheal area ratio may be an available indicator for VR repair surgery. Clinical symptoms and types of VR are heterogeneous and nonspecific in pediatric patients, the diagnosis may be challenging and surgical management is controversial^19^. Some researchers had speculated that the type of VR and presence of tracheal stenosis were important factors in determining the approach to surgery^15,20^. Hong *et al*. reported quantitative evaluation of tracheal stenosis caused by PA sling and assessed the left PA re-implantation outcome^13^. In contrast to the previous reports^13,14^, our data focused on the detailed evaluation of tracheal stenosis in various abnormal forms of VR and we proposed a new parameter to evaluate the degree of tracheal stenosis. The information may be used to determine for VR repair operative intervention while associated other cardiac defects.

Since the Edward’s hypothetical double arch system has been widely adopted to explain the different aortic arch anomalies^21^, knowledge of VR in pediatric has changed substantially in recent decades^22^. In clinical practice, the pattern of arch anomalies, as important information for surgery, can be identified by CT^23,24^. We found diverse types of VR on DSCT, the most common type being the right aortic arch with ALSA and PA sling. These results were different from other reports that consider DAA and RAA with ALSA and left ductus arteriosus to be the most common forms of VR^3,16^. We hypothesized that this difference may be due to the fact that the patients were selected with complications that caused by other cardiovascular malformations, as aortic arch anomalies also can be associated with structural CHD. Kate *et al*. considered that DAA was uncommonly associated with CHD^22^, and RAA with isolated left subclavian artery is associated with CHD in over 50% of cases. As in a previous study^24^, we speculated that PA sling may be more frequently associated with cardiac malformations. Our data were similar to previous reports regarding the detection of uncommon types^4,22^.

As for congenital variants and aortic arch anomalies associated with CHD^3^, previous studies considered that approximately 12% of VRs were associated with cardiac pathology^20^. In our series, TOF was the most common disorder, followed by PDA, and other associated CHD such as atrial septal defect, VSD, and left superior vena cava or more complex malformation.

Our study has several limitations. First, VR presentations vary widely in clinical settings. The types of VR in this study were associated with CHD, implying that selection bias may have existed, and we would include more patients in the further research. Second, as the imaging tests exposed pediatric patients to radiation, we took several measures to reduce radiation dose as much as possible. Finally, a group of VR patients without CHD will be established to clarify the correlation of stenosis severity on DSCT and clinical severity and a long term follow-up proceeding for the prognosis and survival.

In summary, our research further strengthened the belief that DSCT can have an important role in the diagnosis of VR associated with cardiovascular malformations. DSCT scanning not only can accurately display the anatomy of VR in pediatric, but also perform accurate tracheal stenosis measurements that may provide more detailed information for surgery. The tracheal parameter (1-area ratio) might be a feasible and convenient indicator used to assess the surgery in VR patients with CHD.

## Methods

### Study population

A total of 54 patients referred to VR were enrolled from January 2012 to October 2016 retrospectively in our hospital. For the inclusion criteria, the diagnosis of VR was confirmed on DSCT and clinical evidence. Left aortic arch with ALSA was not considered as a type of VR and the PA sling were enrolled^2,3^. The exclusion criteria included the unavailability of imaging materials and incomplete clinical history (n = 5). Forty-nine patients were eligible for inclusion (23 males and 26 females, range 1–120 months), and 13 patients underwent surgical repair of VR. Fifty-six controls without VR and other respiratory disease were enrolled during same period (30 males and 26 females, range 2–122 months), and there are no cardiovascular malformations on DSCT. This study was approved by the Institutional Review Board of West China hospital (No.14-163), and we pledged to abide by the declaration of Helsinki (2000 EDITION) in accordance with the relevant medical research rules of China in the study. Written informed consent regarding radiation exposure and knowledge of adverse reactions to iodinated contrast was obtained from the guardians of all patients prior to CT examination. All patient-sensitive information was treated with strict secrecy and used solely for the purposes of this study.

### Dual-source computed tomography

All examinations were performed using DSCT scanner (Somatom Definition; Siemens Medical Solutions, Forchheim, Germany). A short-acting sedative (chloral hydrate at a concentration of 10%, 0.5 ml/kg) was administered to patients younger than 6 years of age prior to cardiac DSCT examinations; older patients were trained and asked to hold their breath during the scanning.

An Retrospective ECG-gated protocol was performed with the following acquisition parameters: tube voltage of 80 kV, tube current of 100 mAs, gantry rotation time of 0.28 s, and pitch of 0.2–0.5 (adapted to the heart rate, a higher pitch was used for higher heart rates). The ECG-pulsing window was set on Auto. All patients received nonionic contrast agent (iopamidol, 370 mg/ml; Bracco, Italy) at a rate of 1.2–2.5 ml/s via an antecubital vein, followed by 20 ml of saline solution at the same flow rate. The injected volume was adjusted to the body weight (1.5 ml/kg). Scanning was performed in the craniocaudal direction, from the thoracic inlet to 2 cm below the level of the diaphragm. Bolus tracking was used over the region of interest (ROI) in the descending aorta (Ao) with a predefined threshold of 100 HU. Image acquisition was triggered following a delay of 5 s when the ROI attenuation threshold researched 100 HU. All acquired data were processed on a workstation (Syngo; Siemens). The images were reconstructed with a slice thickness of 0.75 mm with an increment of 0.7 mm.

### Image analysis and radiation dose estimation

**Figure 4:**
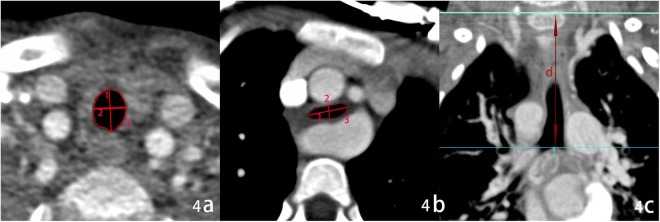
Tracheal measurement on DSCT. (**a**) The tracheal measurement at the level above the aortic arch slice. (**b**) Tracheal measurement at the level of stenosis. (**c**) The total tracheal length measurement (arrow). 1, long diameter; 2, short diameter; 3, trachea area; (**d**) the vertical distance from the upper edge of the first thoracic vertebra to tracheal carina.

For DSCT, anatomic types of VR were classified as follows: right aortic arch, double aortic arch, and pulmonary artery sling (Fig. 1), according to abnormal vessel orientation and tracheal or esophageal compression. The tracheal parameters (short diameter, long diameter, and tracheal area) were measured on the tracheal stenosis (the narrowest site, and the controls around the level of the fourth thoracic vertebra) and above the aortic arch slice (at the level of the second or third thoracic vertebra). The total tracheal length was measured on the three-dimensional reconstruction and represented as the vertical distance, and the total length from the upper edge of the first thoracic vertebra to the tracheal carina (Fig. 4). The degree of tracheal compression was classified as follows: mild (≤25%), moderate (>25%, ≤50%), and severe compression (>50%), depending on the values: 1-long diameter ratio (mean long diameter above the aortic arch divided by tracheal stenosis long diameter, expressed as a percentage), 1-short diameter ratio (mean short diameter above the aortic arch divided by tracheal stenosis short diameter, expressed as a percentage), and 1-area ratio (mean tracheal area above the aortic arch divided by tracheal stenosis area, expressed as a percentage).

Two experienced radiologists recorded these measurements and described morphological characteristics of CHD. One of the experienced radiologists first completed analyzes of all the images and repeated the measurements a week later, another radiologist, who was unaware of these results, reanalyzed the data. The data analysis of tracheal compression measurements were presented as mean value in the research.

The volume CT dose index (CTDIvol) and dose-diameter product (DLP) were automatically displayed on the CT console after examination. The effective dose (ED) was calculated by multiplying the conversion coefficients according to the 2015 recommendations of the International Commission on Radiological protection^25^.

### Clinical material

Cardiovascular malformations were classified and the surgeries were recorded. The respiratory manifestations of patients were graded from mild to severe based on the number of hospital admissions^26^. The extremely severe referred to more than 4 admissions and associated with tracheal dysplasia such as bridging bronchus.

### Statistical analysis

Statistical analysis was performed using SPSS software (version 21.0 for Windows; SPSS, Chicago, IL, USA) and MedCalc software (version 15.0, MedCalc Software; Mariakerke, Belgium). Continuous variables were expressed as mean ± standard deviations, and categorical variables were expressed as numbers and percentages. The independent sample t-test was used to compare the tracheal stenosis between the VR pediatrics with CHD and controls. The Mann-Whitney U test or Fisher’s exact probability test were used for comparing the VR surgery among three degree of tracheal compression. Bland–Altman analysis was performed to assess the agreement between (1-long diameter ratio) and (1-area ratio), and (1-short diameter ratio) and (1-area ratio) in patients and controls, respectively. And Pearson’s correlation analysis was used to assess the correlation between the degree of tracheal compression and respiratory manifestation grade. Intra-class correlation coefficients (ICCs) were calculated to assess the intra- and inter-observer variability. A two-tailed P value of <0.05 was considered statistically significant in all analyses.
